# Clinical Outcomes of Telemonitoring for Patients on Warfarin after Discharge from Hospital

**DOI:** 10.1155/2018/7503421

**Published:** 2018-08-12

**Authors:** Natthaporn Sudas Na Ayutthaya, Itsarawan Sakunrak, Teerapon Dhippayom

**Affiliations:** ^1^Department of Pharmacy, Kamphaeng Phet Hospital, Mueang Kamphaeng Phet, Thailand; ^2^Faculty of Pharmaceutical Sciences, Naresuan University, Phitsanulok, Thailand

## Abstract

**Objective:**

To evaluate the impact of telephone follow-up service on clinical outcomes in patients on warfarin when discharged from hospital.

**Methods:**

This randomized controlled trial was conducted at a general hospital in Thailand. Patients aged ≥20 years who were prescribed warfarin when discharged were eligible to participate in this study. They were randomly allocated, using a computer generated random number, to receive either telephone follow-up intervention or usual care. Participants in the intervention group received telephone follow-up by hospital pharmacists for three months. During each telephone call, pharmacists performed medicine use reviews and addressed any problems identified.

**Key Findings:**

A total of 50 patients participated in this study. The proportion of international normalized ratio (INR) values in the target range for the telephone follow-up group (36/79, 45.6%) was higher than that in the usual care group (19/79, 24.1%), p=0.005. The mean time in the therapeutic range (TTR) in the telephone follow-up group was also higher than that in the usual care group (49.8±34.3 versus 28.0±27.5, p=0.017). All patients in the usual care group experienced one or more out-of-range INR values (25/25, 100%) compared to 21 out of 25 (84%) in the telephone follow-up group, p=0.037. There was no difference between the two groups in the incidence of complications or adverse events associated with warfarin.

**Conclusions:**

The telephone follow-up service in recently discharged patients helps them achieve and maintain their INR target. This anticoagulant supportive service should be promoted to patients receiving warfarin therapy after discharge. This trial is registered with TCTR20180614006 (Thai Clinical Trials Registry).

## 1. Introduction

Warfarin is a well-established oral anticoagulant used for the prevention and treatment of thromboembolism and thromboembolic complications in patients with atrial fibrillation, heart valve replacement, or myocardial infraction [1]. Due to its narrow therapeutic index and dosage variability among patients, individuals receiving warfarin therapy require dose adjustment based on the international normalized ratio (INR) to reduce the risk of adverse reaction such as thrombotic and bleeding events that could lead to hospitalization or life-threatening conditions [2].

Several studies have demonstrated that pharmacist-managed anticoagulation clinics improve time in therapeutic range, lower the incidence of adverse events, and reduce the need for frequent office/anticoagulant clinic visits [3]. Anticoagulant management can be achieved through office visits or telephone visits, which proved to be comparable [4–6]. The advantages of telephone-based management of warfarin are that it provides time and cost savings, increases access to care, provides convenience, and reduces the risk of anticoagulation therapy related complications. Homebound patients have also reported a high degree of satisfaction with telephone-based anticoagulation management [7]. However, anticoagulant management via telephone calls might not be feasible everywhere as it requires INR measurements, and healthcare structures in some areas may not support the implementation of this service due to limited access to INR measurement facilities outside hospital settings. A previous study revealed that continuous warfarin follow-up counseling through phone calls and home visits helped improve INR control in patients discharged on warfarin [8]. However, the intensive intervention employed in this study, i.e., weekly telephone and fortnightly home visit for three months, may not be a practical approach for many settings. Also, telephone-based management of warfarin is deemed suitable for patients with stable INRs, while those with an unsteady INR may require alternative approaches.

Hospitalization is one of the potential causes of INR fluctuations in the postdischarge period, particularly among patients who newly initiated therapy [9]. Patients who are discharged on warfarin require close follow-up monitoring to ensure the safe and effective use of warfarin as their clinical conditions may not be as stable as ambulatory patients. Telephone follow-up adds another layer of service; currently, no evidence exists to justify that a telephone follow-up service could help patients achieve their INR target. The objective of this study is therefore to evaluate the impact of telemonitoring using a telephone follow-up service on clinical outcomes in patients on warfarin after discharge from a hospital.

## 2. Methods

This study is a randomized controlled trial with parallel group design and a 1:1 allocation ratio. It was conducted between May and September 2016 in Kamphaeng Phet Hospital, a 410 bed general hospital in the Lower Northern Region of Thailand. The study protocol was approved by Naresuan University Institutional Review Board (NU-IRB no. 119/59). Informed consent was obtained from all patients before their participation in this study.

### 2.1. Participants

Patients aged 20 years or older were eligible for inclusion if they (i) were prescribed warfarin upon discharge, (ii) had no scheduled surgery within three months after discharge, (iii) were able to manage their own medication, (iv) could communicate in Thai, and (v) possessed a telephone. Those who were prescribed warfarin prior to admission and had INR values in target ranges throughout the preceding three months were excluded.

As no similar study was found that could be used for sample size calculations, the number of participants in this study was based on a recommendation by Collins et al. for experimental research design [10]. Accordingly, a minimum of 21 participants per group was needed to detect statistically significant differences in one-tailed and/or two-tailed tests with 80% power and a 5% alpha level. Taking into account a predicted dropout rate of 20%, the number of participants per group in this study was 25.

### 2.2. Interventions

Participants in the telephone follow-up group and the usual care group received standard pharmacy services for patients discharged on warfarin, which included (i) assessing appropriateness of the warfarin dose, (ii) checking for warfarin drug interaction with other concomitant medicines, (iii) educating patients about the indication and the use of warfarin, (iv) assessing and identifying factors associated with warfarin adherence for those who had been using warfarin prior to admission, and (v) checking for patients' understanding of the given information.

Three clinical pharmacists who worked in medical wards were informed about the telephone follow-up procedures and were checked for their fidelity to the study protocol on a monthly basis. After discharge of the participants in the telephone group, pharmacists made one phone call before the next in-person follow-up based on the following schedules: day three for a one-week scheduled visit, day seven for a two-week scheduled visit, and day fourteen for a three- or four-week scheduled visit. For the second and subsequent visits within the three-month study period, pharmacists continued to make telephone calls on a regular interval as follows: day three for a 1-week scheduled visit; days three and seven for a two-week scheduled visit; days three, seven, and fourteen for a three-week scheduled visit; and days seven, fourteen, and twenty-one for a four-week scheduled visit.

During each 10–25-minute telephone call, pharmacists performed medicine use review by asking patients about problems/obstacles with managing warfarin including adverse events and complications, assessing medication adherence, and giving reminders for the next scheduled visits. Pharmacists promptly addressed any problems identified during the telephone call with patients.

### 2.3. Outcomes and Data Collection

The primary outcomes were the proportion of INR values in range, time in therapeutic range (TTR), proportion of INR out of range, and the number of patients with one or more out-of-range INR values. Safety outcomes were the frequency of complications or adverse events associated with warfarin, i.e., bleeding events, thromboembolic events, emergency room visits, and hospitalization. Bleeding in the following locations was classified as major bleeding: intracranial, intraspinal, intraocular, retroperitoneal, intra-articular, pericardial, and intramuscular [11]. Other complications included depletion of hemoglobin to 1.24 mmol/L or less and bleeding that required more than 2 units of blood transfusions.

All INR testing was performed with ACL TOP 300 CTS machine, which was calibrated by technicians from the instrument's company every month. The TTR was calculated with the Rosendaal method using INR measurements from all warfarin clinic visits for each patient. The medical technologists were not aware of the study groups when they performed and reported the participants' INRs.

Demographic characteristics, indication for warfarin therapy, and goal INR were extracted from medical records for each patient. Warfarin clinic records were the source of data on outcomes of interest. At each clinic visit, patients were asked to report complications/adverse events such as bleeding and thrombosis. Pharmacists at the warfarin clinic then checked for signs and symptoms of reported safety outcomes. Participants were also asked to report any emergency room visits or hospitalization, which was subsequently confirmed with hospital records.

### 2.4. Randomization

The study population was stratified by experience of using warfarin, i.e., individuals who had used warfarin before admission and naïve users who were first prescribed warfarin during the course of the current admission. A random allocation sequence was created using a computer generated random number table. Random numbers were subsequently put into opaque envelope and sealed. Clinical pharmacists who worked on medical wards identified eligible patients and obtained signed informed consent before they were discharged. During the recruitment phase, information about newly enrolled participants was sent to the principal investigator to assign participants to an intervention.

### 2.5. Statistical Analysis

Participant demographic characteristics were compared using either the chi-squared test for nominal data or the* t*-test for continuous data. All INR measurements, except measures after readmission, in each group were used to calculate the percentage of INR values in therapeutic and out of therapeutic range (subtherapeutic and supratherapeutic). The chi-squared test was used to compare the difference in INR values in therapeutic range, INR values out of range, patients with one or more out-of-range INR values, and safety outcomes. TTR was calculated from INR value records for each patient during the study period. INR records after readmission were not used for TTR calculations. The overall mean TTR in both groups was compared using a* t*-test. All statistical analyses were performed using SPSS, version 17.0; SPSS Inc.; Chicago, IL). A p-value of <0.05 was used to determine statistical significance.

## 3. Results

**Figure 1:**
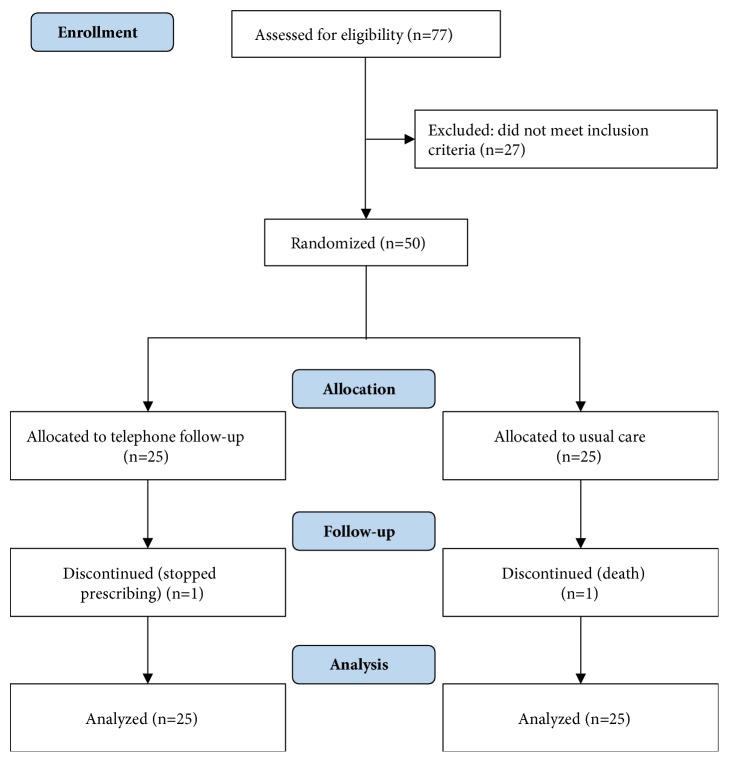
CONSORT flow diagram.

The recruitment period took place between May and June 2016. Once enrolled, each participant was followed for three months with the last follow-up ending in September 2016. A total of two, out of fifty, participants did not complete the study. One participant from the telephone follow-up group no longer required warfarin due to an improvement in indicated condition (deep vein thrombosis), while one individual from usual care group died (Figure 1).

The majority of participants in the telephone follow-up group were female (18/25, 72%), which was different in the usual care group (12/25, 48%). Mean age of participants in both groups was not different, i.e., 56.6±11.9 years and 58.7±10.0 years in telephone follow-up and usual care group, respectively. Participants who were prescribed warfarin for the first time accounted for half of the participants in each group (13/25, 52%). The main indication for warfarin in both groups was atrial fibrillation, i.e., 56% in the telephone follow-up group and 68% in the usual care group. Other prescribed indications included valvular heart disease, mechanical prosthetic valves, deep vein thrombosis, and pulmonary embolism. Participants in both groups shared common comorbidities such as diabetes, hypertension, and dyslipidemia. Almost all participants had the same target INR of 2.0-3.0. Only two (8%) participants in the telephone follow-up group and one (4%) in the usual care group had their INR goal at 2.5-3.5 (Table 1).

Pharmacists made a total of 179 successful telephone calls to participants in the telephone follow-up group with an average of 7.16 calls for each participant (range 7–10 calls). During the three month study period, participants in both groups had their INR measured on average 3.4 times. Specifically, a total of 79 INRs were measured in each group. Thirty-six INRs (45.6%) were in range for the telephone follow-up group, whereas only 19 INRs (24.1%) in the usual care group had INR values in the target range, p=0.005 (Table 2). The mean (standard deviation) TTR in the telephone follow-up group was also higher than that in the usual care group, 49.8 (34.3) versus 28.0 (27.5), p=0.017. For out-of-range INR values, the proportion of supratherapeutic INR values in the telephone follow-up group (9/79, 11.4%) was significantly lower than those in the usual care group (19/79, 24.1%), p=0.037. However, this was not the case for subtherapeutic INR values as the proportions of subtherapeutic INRs in the telephone follow-up group and the usual care group were not different, 34/79 (43.0%) versus 41/79 (51.8%), p=0.265. When taking into account the number of patients experiencing one or more out-of-range INR values, it was found that all participants in the usual care group had at least one previous record of an INR value out of target range (25/25, 100%) compared to 21 out of 25 (84%) in the telephone follow-up group, p=0.037.

Complications reported in both groups were not different. A total of three major bleeding events occurred during the study period; two were from the telephone follow-up group, and the other from the usual care group. Although not statistically significant, thromboembolic events and warfarin-related hospital admissions in the telephone follow-up group seemed to be relatively lower than those in the usual care group, 3/25 (12%) versus 6/25 (24%), p=0.259, and 4/25 (16%) versus 7/25 (28%), p=0.306, respectively (Table 3).

## 4. Discussion

To the best of our knowledge, this is the first study in the field using a RCT design to evaluate the impact of a telephone follow-up service in patients on warfarin. Hospitalized patients who were discharged on warfarin and received the telephone follow-up service had a higher rate of INR in target range and a higher TTR percentage than those who received usual care. The telephone follow-up service also decreased the number of patients experiencing out-of-range INRs after hospital discharge. This study also proved that the service is safe for patients as both study groups experienced rare and similar rates of thromboembolic and bleeding complications.

Most previous studies [4–6] explored the effects of telephone interactions for anticoagulant management based on INR readings, while the telephone follow-up in this study focused on medicine use review to evaluate issues in managing warfarin and to assess medication adherence. This may explain why this telephone intervention showed improvement in the proportion of INR values in range and TTR percentage while telephone-management services in previous studies were comparable to standard warfarin management [4–6]. Another explanation is that most patients in previous studies had consistently stable INR values; hence, it would be difficult to see any additional benefit in these patients.

The beneficial effects of a telephone follow-up service found in this study are well aligned with findings from Ibrahim et al. which showed that follow-up counseling through phone calls every week and home visit biweekly for three months improved INR control [8]. However, the present study demonstrated that telephone follow-up alone without home visits is sufficient to help patients achieve and maintain their INR target. This telephone follow-up approach might be more practical and cost effective than the combination of telephone and home visits in patients discharged on warfarin, although it requires a head-to-head comparative trial to explore which intervention is more effective.

Despite patients who received telephone follow-up service having a higher percentage of TTR compared with those in the usual care group, they were only at goal 44% of the time, which does not indicate a good response to warfarin management, i.e., 60% or more [12]. A number of potential factors have been shown to affect discharge INRs including new initiation of warfarin therapy on hospital admission, shorter hospital stay periods, and concurrent infection [13]. Current recommendations for the initiation of warfarin therapy are 3–5 mg per day although the initial dose could be lower in some patients such as elderly patients, patient with increased risk of bleeding, or patients with liver disease [14, 15]. In this study, warfarin initiation doses prescribed in both groups were lower than current recommendations due to participants' comorbidities and complications prior to admission. This could explain the relatively low TTR and high proportion of subtherapeutic INR values that were observed in this study. It was also suggested that the TTR measurement is not recommended for the first month after initiating warfarin [16]. Using INR measurements for the first month after discharge for patients whose clinical conditions were unstable may reduce the calculated TTR as found in this study.

There are some limitations in this study. First, the duration of the telephone follow-up period was only 3 months, which may not be sufficient to observe effects that require long-term follow-up such as death or rehospitalization related to warfarin therapy. The sample size in this study also appeared relatively low compared with other trials. Nonetheless, it was adequate to statistically identify the difference in INR values in range and TTR between the telephone follow-up and usual care groups. The limitation of this small sample size was that it restricted the ability to undertake further subgroup analysis. Another limitation is that patients were recruited from one hospital, which may not represent the general population. It should be noted, however, that several characteristics of these patients including age, comorbidities, warfarin indication, and target INR suggest that they are comparable with general patients who receive warfarin after discharge.

## 5. Conclusion

This randomized controlled trial demonstrated that a telephone follow-up service when combined with usual care in recently discharged patients on warfarin helps patients achieve and maintain their INR target. Further studies with more participants and longer study periods may be needed to explore the impact of this service on complications of warfarin therapy. This anticoagulant supportive service could be a feasible approach to improve clinical outcomes and should be promoted to patients receiving warfarin therapy after discharge.

## Figures and Tables

**Table 1:** Demographic characteristics.

**Characteristics**	**Telephone (n=25)**	**Usual care (n=25)**	**p-value**
Gender, female	18 (72%)	12 (48%)	0.083
Age, years	56.6+11.9	58.7+10.0	0.506
Experience of using warfarin			
Naïve	13 (52%)	13 (52%)	1.00
<3 month experienced	5 (20%)	3 (12%)	0.440
>3 month experienced	7 (28%)	9 (36%)	0.544
Indication			
Valvular heart disease	6 (24%)	8 (32%)	0.529
Mechanical prosthetic valves	2 (8%)	1 (4%)	0.552
Atrial fibrillation	14 (56%)	17 (68%)	0.382
Deep vein thrombosis	8 (32%)	6 (24%)	0.529
Pulmonary embolism	0 (0%)	1 (4%)	0.312
Comorbidities			
Diabetes	4 (16%)	5 (20%)	0.713
Hypertension	9 (36%)	15 (60%)	0.089
Dyslipidemia	7 (28%)	8 (32%)	0.758
Ischemic heart disease	3 (12%)	1 (4%)	0.297
Heart failure	5 (20%)	6 (24%)	0.123
Chronic kidney disease	5 (20%)	6 (24%)	0.733
Others	3 (12%)	2 (8%)	0.637
Warfarin dosing on discharge (mean+SD)	18.2±6.7	18.1±4.6	0.922
Target INR			
2.0-3.0	23 (92%)	24 (96%)	0.552
2.5-3.5	2 (8%)	1 (4%)	0.552

INR: international normalized ratio.

**Table 2:** Therapeutic outcome measures.

**Outcomes**	**Telephone **	**Usual care **	**p-value**
INR values in range^†^	45.6% (36/79)	24.1% (19/79)	0.005
TTR^‡^	49.8+34.3	28.0+27.5	0.017
Out of range INR values			
Subtherapeutic^†^	43.0% (34/79)	51.8% (41/79)	0.265
Supratherapeutic^†^	11.4% (9/79)	24.1 % (19/79)	0.037
Patients with >1 out of range INR value	84.0% (21/25)	100.0% (25/25)	0.037

^†^Percentage of INR values out of all INR measures. ^‡^Mean + standard deviation. INR: international normalized ratio. TTR: time in therapeutic range.

**Table 3:** Complications.

**Outcomes**	**Telephone (n=25)**	**Usual care (n=25)**	**p-value**
Bleeding events			
Major bleeding	2 (8%)	1 (4%)	0.552
Minor bleeding	3 (12%)	4 (16%)	0.684
Thromboembolic events	3 (12%)	6 (24%)	0.259
Warfarin-related hospital admission	4 (16%)	7 (28%)	0.306

## Data Availability

The data used to support the findings of this study are available from the corresponding author upon request.
